# How can the Sendai framework be implemented for disaster risk reduction and sustainable development? A qualitative study in Iran

**DOI:** 10.1186/s12992-024-01028-w

**Published:** 2024-03-21

**Authors:** Mahmood Nekoei-Moghadam, Seyed Mobin Moradi, Asghar Tavan

**Affiliations:** 1 Health in Disasters and Emergencies Research Center, Institute for Futures Studies in Health, Kerman University of Medical Sciences, Kerman, Iran; 2 Faculty of Management and Medical Information Sciences, Kerman University of Medical Sciences, Kerman, Iran

**Keywords:** Sendai framework, Disaster risk reduction, Developing countries, Executive actions, Strategies

## Abstract

**Background:**

The Sendai Framework is the United Nations’ most significant approach to reducing the risk of disasters from 2015 to 2030. This framework designed for all communities. However, communities should create operational and remedial strategies based on their unique circumstances. Considering the gaps in the implementation of Sendai framework strategies in Iran, as a developing country, the present study was designed.

**Method:**

This study was conducted by using a qualitative direct content analysis method to find out the expert’s opinions on the implementation of the Sendai framework in Iran from 2021 to 2023. 35 experts in the focus group discussion and 9 experts in the interview were the participants of the study.

**Results:**

Study findings were merged and reported as one main theme entitled Executive actions for implementing the Sendai Framework, four categories, and 37 codes. Eleven codes for the strategy of understanding disaster risk, 11 codes for the strategy of strengthening disaster risk governance to manage disaster risk, eight codes for the strategy of Investing in disaster risk reduction for resilience, and finally, seven codes for the strategy of enhancing disaster preparedness for effective response and to “Build Back Better” in recovery, rehabilitation, and reconstruction were identified as implementation solutions.

**Conclusion:**

The Sendai Framework has not provided any detailed implementation solutions because the countries’ economic, social, level of development, etc., are different. The study’s findings can be used as a guide for other developing countries.

## Background

The concept of sustainable development has evolved into a paradigm for development and has become a reference for environmental science study [39]. Sustainable development is much more than economic development and includes wide dimensions such as social, economic, political, cultural, environmental, and local and international policies [16]. Due to their different conditions, developing and developed countries have different approaches and obstacles to achieving sustainable development goals [38]. The occurrence of disasters is one of the main obstacles to achieving sustainable development goals. Disasters lead to spending society’s resources to recover the affected areas, and opportunities to invest in sustainable development goals are lost [7]. In this way, investing in disaster risk reduction can be one of the available options for vulnerable communities to achieve sustainable development goals [18].

It is rare to find a society in the world that does not face various types of disasters, whether natural or man-made [8]. Today, disasters have taken up a large part of the government’s resources and programs [47]. Climate changes, human manipulations in nature and the rapid development of technology have increased the vulnerability of people and the occurrence of accidents [48]. The statistics show the increasing growth of disasters all over the world. Disasters impose many lives and financial losses on governments and nations every year [28].

Statistically, the Asian continent has faced the most natural disasters, 44%, and also the most affected people, 69.5% of the disasters, were from Asia. After that, the Americas with 25%, Europe with 14%, Africa with 13% and Oceania with 4% are in the next rank [31]. Therefore, it can be seen that the Asian continent is one of the most vulnerable continents in terms of the number of disasters and the number of people killed. Meanwhile, Asia and Africa are the most vulnerable [32]. On the other hand, it is estimated that more than 97% of natural disasters that lead to death, occur in developing countries that are located in disaster-prone areas. In addition to natural disasters, man-made disasters also occur in developing countries due to inadequate infrastructure [25]. A study on the future impacts of disasters estimates that between 2020 and 2030, people in Asia and the Pacific will be at high risk for injury and death from various disasters [31]. On average, accidents and disasters cause more than 150,000 lives and more than 140 billion dollars in losses in the world’s countries every year [34].

The prevention and mitigation of disaster risk must be the primary concern for all parties involved, including managers, experts, and politicians. Utilizing global information and experiences might be a helpful step in raising the degree of catastrophe knowledge in this regard. The Sendai Framework is the United Nations’ most significant approach to reducing the risk of disasters from 2015 to 2030 [36]. The Sendai Framework comprises parts that guarantee and support continuous efforts by governments and other stakeholders. Many experts believe that the following modifications to the Sendai framework are the most significant: a focus on disaster risk management instead of crisis management [35].

The Sendai Framework is a fully structured framework with particular objectives and plans for every community. However, it is up to the communities to carry it out. Communities should create operational and remedial strategies based on their unique circumstances [11]. In their study, Chisty et al. examined the compliance of disaster management policies in Bangladesh with the Sendai framework strategies. According to their findings, Bangladesh has been able to align disaster management policies and programs with the priorities of the Sendai Framework since 2015, but there are still areas for improvement. The most progress has been in the priorities of recognizing and understanding disaster risk and strengthening governance and leadership for disaster risk management, and strategies for investing in disaster risk reduction and promoting disaster preparedness still need improvement. They state that strategy implementation methods should be designed with the participation of stakeholders [8]. In the study of Wei et al., the effect of the Sendai Framework on construction regulations and codes in Australia has been investigated. The findings show a statistically significant connection between building flexibility, the Sendai Framework, and national construction legislation. They also stress that disaster patterns vary across countries. Risk analysis studies should be conducted to ensure the resilience of cities and communities against disasters. Each society should adapt its policies to the Sendai document based on existing risks and needs [51]. In 2020, Wright et al. conducted a study entitled “Managing Health Emergencies and Disasters: Five Years of Implementation of the Sendai Framework.” According to this study, the Sendai Framework for Disaster Risk Reduction places health at the center of DRM at the international policy level. After five years, this framework has led to the development of the health sector in risk management through intersectoral partnerships and increased scientific research [53]. Aitsi-Selmi et al. state that the Sendai framework for disaster risk reduction represents a step towards global policy coherence with explicit reference to health, development, and climate change. They state that to reduce risks, the United Nations Office for Disaster Risk Reduction (UNISDR) has organized several global, regional, national, and intergovernmental technical meetings, and it has proposed progress criteria to reduce the risk of disasters to the member countries. However, the suggested items are general, and their implementation is assigned to the countries. In this way, each country can design and implement policies based on its cultural, economic, social, etc., priorities and conditions [2].

Iran, as a developing country, is one of the most vulnerable countries in the world, as it has suffered extensive losses (human and financial) due to the occurrence of such disasters in the past few decades (Boyen Zahra earthquake, 1963 to Bam earthquake, 2003) [12]. Due to the variety of weather and strategic geographical location and being on the border with various Asian, European, and Arab countries, it has always been popular with tourists and immigrants from other countries. Iran has an area of 1,648,195 square kilometers and is located on the Alpine-Himalayan earthquake belt; it is always at risk of earthquakes caused by the activation of faults (Zarand and Bam earthquakes are notable examples). 77% of 300 populated cities have earthquake faults, and 35% of Iranian towns are exposed to floods, storms, and coastal waves [45]. Over the past few years, implementing Sendai framework strategies in Iran has faced many challenges and problems, including economic, social, and cultural issues. On the other hand, the Covid-19 pandemic led to a change in priorities, and the strategies of this framework were neglected more than before. As seen in various studies, the importance of adaptation and coordination of Sendai framework strategies with political, social, economic, and cultural features has been emphasized a lot. Considering the gap in the implementation of Sendai framework strategies in Iran, as a developing country, the present study was designed and implemented with the aim of “How can the Sendai framework be implemented for disaster risk reduction in Iran?”. As mentioned before, there is no single solution for implementing the Sendai Framework in different societies; however, from the results of this study, many challenges in implementing the Sendai Framework in developing countries can be identified, and their solutions can be understood.

## Method

### Study design

This study was conducted by using a qualitative direct content analysis method to find out the expert’s opinions on the implementation of the Sendai framework in Iran from 2021 to 2023. The directed content analysis is a methodology that explores a phenomenon of interest using a theory as a guide [17]. The framework for this study is the main Sendai framework strategies. By means of this methodology, the researchers explored the opinions of the participants, which included solutions for the implementation of the Sendai strategies in the country. This manuscript was prepared in accordance with the COREQ standards for qualitative research reports.

### Participants

In this study, according to the purpose of the research, a focused group discussion was first conducted to collect data. Then, a semi-structured face-to-face interview was conducted to obtain data from different sources (Triangulation).The participants in this study were selected by a purposive method. The sample of the present study was selected in a focused group discussion and included 35 experts from different fields of risk management. The participants were between 35 and 72 years old; 11 members were female, and 24 were male. People participating in the group discussion included experts of health in disasters (8 people), health service management (4 people), health economics (1 person), health policy makers (3 people), risk experts (geological, water and air, man-made) (6 people), emergency medicine specialist (3 people), social medicine (2 people) and crisis management experts at the national, provincial and local levels (8 people). The inclusion was having experience in risk management, knowledge of disaster risk reduction, especially the Sendai framework, and interest in participating in the study. The interviews were conducted face-to-face, and sampling continued until data saturation using the purposive and snowball method so that no new data was obtained by conducting more interviews. At this stage, there are nine people, including health in disasters (2 people), a risk expert (2 people), a health policy specialist (1 person), a health management specialist (1 person), and an emergency medicine specialist (1 person). Finally, the key people of crisis management at the national level (2 people) participated.

### Data collection

To collect data in this study, in addition to demographic profile questions, a semi-structured questionnaire based on action priorities in the Sendai framework was used. At first, the facilitator explained the study goals and ethical considerations, then started the discussion session with a general question about the obstacles to implementing the Sendai framework, and then directed the questions to the obstacles to implementing action priorities. The meeting lasted 240 min in an academic center. The corresponding author managed the meeting as a facilitator, and two other authors contributed to this meeting as data recorders. FGD was recorded in mp3 and was transcribed by the research team within two days. Field notes were also made at the same time. In the individual interview, the researcher was looking for more transparency and completion of corrective solutions using the data extracted from the previous stage and the semi-structured interview. The interviews were conducted at the participants’ workplace and lasted 40 ~ 70 min. The interview questions were developed in discussion with qualitative research experts and based on the main priority of the Sendai Framework and focus group discussion. The interview questions were pilot-tested.

### Data analysis

The study’s data analysis followed the suggestions provided by Hsieh and Shannon for directed content analysis. Directed content analysis was performed when a framework or prior research exists about an incomplete phenomenon or would benefit from further description. The qualitative researcher might choose to use a directed approach to content analysis. The steps of this approach are such that after specifying the analysis unit, coding by assigning a code to each meaning unit and placing codes with similar meaning in the subcategories and main categories and the main themes of semantic units are identified [17]. Two authors conducted data coding. In this study, the four main strategies of the Sendai framework were taken into consideration as the default main categories. The codes and subcategories from the date were placed in this format. MAXQDA Ver-2020 software was used to analyze the qualitative data.

### Strategies to achieve rigor in the study

Lincoln and Guba’s evaluation methods were used to ensure the validity and reliability of the study, which are equivalent to the validity and reliability of quantitative research. Based on this method, four criteria of validity, transferability, reliability, and verifiability were considered for evaluation [14]. The following steps were taken to achieve these criteria: Validity: Sufficient time was spent to conduct this research, and four experts approved the research process. In addition, to ensure valid coding, two independent coders were used to code several interview samples. Transferability: To ensure the transferability of research findings, three experts who were not involved in the research were consulted on the research findings. To ensure the transferability in the data analysis stage, special coding and symbol analysis procedures were used. Research details and notes were recorded at all stages of the work to ensure reliability. Verifiability: If the research findings are verifiable, all details must be carefully recorded at all stages, which was done for the present study. In addition, all data, notes, documents and recordings were kept for possible further reviews.

## Results

After extracting 1000 initial codes from the focus group discussion and individual interviews, these codes were merged and reported as one main theme, four categories, and 37 codes. These results are reported in Table 1. The main theme of this study was to explain the implementation strategies of the Sendai Framework for Disaster Risk Reduction in Iran as a developing country. The Sendai framework states that to achieve the goal of disaster risk reduction, it is necessary to pay attention to four strategies (Understanding disaster risk, strengthening disaster risk governance to manage disaster risk, investing in disaster risk reduction for resilience and enhancing disaster preparedness for effective response and to “Build Back Better” in recovery, rehabilitation and reconstruction) is essential. These four strategies were the main categories of the study. Codes that might be used to implement the Sendai Framework were found for every main category. The extracted categories and codes are described in the paragraphs that follow.

**Table 1:** Theme, categories and final codes of the study

**Main theme**	**Categories**	**Codes**
**Executive actions for the implementation of the Sendai framework**	**Executive actions for implementing the Sendai framework in the strategy of Understanding disaster risk**	1. analyzing risk history and creating a risk zoning map 2. Establishing and enhancing disaster research institutes, as well as their networking 3. Utilizing dependable and scientific techniques to identify, comprehend, and evaluate risks 4. Development and introduction of native risk comprehension tools based on the nation's resources 5. Employing elites from many professions to form specialized risk recognition groups and to help develop a common terminology 6. Establishing a national, supra-factional organization or giving authority to an existing organization to get the support and involvement of high-ranking public and private sector managers 7. Establishing integrated information infrastructures to identify risks, define them, and evaluate possible threats for them 8. Integrating risk management into organizational missions with regard to sustainable development goals 9. Developing educational programs to promote Society risk understanding and localize it 10. Fostering a culture of risk awareness and understanding at all societal levels through the use of mass and personal media 11. Focusing on non-governmental organizations (NGOs) to raise societal awareness
	**Executive actions for implementing the Sendai framework in the strategy of strengtheningdisaster risk governance to manage disaster risk**	1. Combining related organizations with the same functions to form a single national organization 2. Establishment of regional risk governance laws and regulations, as well as the development of implementation and facilitation measures for these laws 3. Strengthening risk management's position in governance documents by including it in 5-year plans 4. Stakeholder analysis to get approval, offer dependable funding, and improve the collaborative strategy of stakeholder organizations 5. Establishing an information network at the national level for distributing information to relevant entities 6. Compilation of requirements for obtaining a risk management position following the meritocracy principle 7. performing comparative research to discover a model for country and standardizing rules and procedures 8. the creation of an extensive risk management strategy, regular and ongoing national monitoring, and a focus on being program-oriented rather than individualistic 9. Using expert managers in risk management and improving their scientific level 10. Increasing the proportion of funds for risk reduction in the national macro budget 11. Attention on comprehensive risk management, including risk probability, capacity, and vulnerability.
	**Executive actions for implementing the Sendai framework in the strategy of Investing in disaster risk reduction for resilience**	1. Utilizing legislative tools (such as tax breaks, loan grants, etc.) to encourage individuals to invest in this sector while explicitly communicating the difficulties resulting from disasters to them 2. Training to managers and community influencers to encourage investment in DRR 3. Incorporating DRR projects into current construction and development programs and allocating funding from the National Development Fund 4. Conducting comparative research to draw on global experiences regarding the effects of an investment in DRR and access to technical and transparent disaster information 5. Development of knowledge-based companies in the field of DRR and investment in it 6. Approval of laws for the investment of economic enterprises, polluting industries and insurance in DRR and revision of building regulations. 7. Preventing the advertising of harmful products and services and promoting the purchase of safe products, personal safety equipment, and other necessary safety and protection equipment by the general public 8. Adopt legislation to compel governments to invest in DRR and facilitate the participation of other sectors such as NGOs, private and charitable institutions, and international organizations.
	**Executive actions for implementing the Sendai framework in the strategy of enhancing disaster preparedness for effective response and to “Build Back Better” in recovery, rehabilitation and reconstruction**	1. Establishing an up-to-date early warning system and designing community-based early warning protocols 2. Developing and enhancing the incident command system to support and coordinate actions across agencies responsible for disaster response. 3. Compilation of a comprehensive disaster preparedness plan and practice the program in regular intervals. 4. Preparation and compilation of training programs for disaster preparedness and response according to the target groups (from elementary schools to households and universities). 5. Paying special attention to the role of social networks and media in transmitting timely and correct information during disasters and providing proper education to the people 6. Improving information systems and technologies, including simulation, GIS, etc., to develop risk maps, forecast disasters, logistics, etc. 7. Use of NGOs, private sector and international organizations to increase preparedness in society.

### Executive actions in the strategy No1: understanding disaster risk

This strategy has identified some solutions to address potential risks, which include adhering to scientific principles to identify risks and finding effective ways to share this information with stakeholders. To put this strategy into action, 11 executive actions have been identified. One important aspect highlighted by experts is the need to analyze risk history and create a risk zoning map. Experts emphasized that understanding the risks in each region and creating a map of those risks can improve the decision-making process. An expert said, “Creating regional maps based on existing risks can save time and cost. However, it’s important to note that these maps need to be reviewed and updated regularly.” (P-9). Another issue in this field was establishing and enhancing disaster research institutes and their networking. An expert said, “One solution to monitor risks and develop suitable solutions is establishing regional research centers where experts can conduct scientific research. These centers must be connected so they can share their experiences and knowledge. This collaboration can improve the effectiveness of their research and ultimately lead to better risk management strategies.” (P-3). Using reliable and scientific methods to identify, understand, and evaluate risks is crucial. The experts stressed the importance of using modern and appropriate techniques based on the economic and cultural conditions of the society. This will ensure a better understanding and recognition of potential risks. An expert said, “There are several methods for risk recognition and analysis. We can identify these methods, learn them, and use them based on our condition.” (P-28). The development and introduction of native risk understanding tools based on the nation’s resources was recognized by experts as a critical issue. One of the participants said, “The current tools we use to measure risks are not specific to our country and do not consider unique cultural and social conditions. It is therefore recommended to produce native tools that are tailored to our country’s needs. This would make it easier and more logical for both the evaluator and the analyst to understand and analyze the results.” (P-33). Another solution proposed in this area is to employ professionals from different fields to form specialized groups for risk recognition and to aid in developing a common terminology. An expert said, “I believe that if working groups at the national level with diverse expertise are formed to evaluate different regions and present the results in a simple and understandable language for the public, many issues related to risk perception can be resolved.” (P-1). Another solution emphasized by the experts was establishing a national, non-partisan organization or granting authority to an existing organization, with the support and involvement of high-ranking officials from both the public and private sectors. They stated that the existence of multiple organizations with parallel tasks can lead to a waste of resources. An expert said, “One of the major issues in crisis management is the existence of multiple structures with overlapping responsibilities. It is suggested that instead of designing a new organization and creating additional bureaucracy, the current structures should be reorganized to assign the task of identifying risks to a specific department” (P-3). Another solution identified in this category is the establishment of integrated information infrastructures. These infrastructures aim to identify risks, define them accurately, and evaluate potential threats associated with those risks. An expert said, “If our systems are outdated and lack the proper infrastructure to record risks in real-time, it’s natural that there will be delays in presenting data, which can result in a loss of effectiveness” (P- 13). Another study result was integrating risk management into organizational missions about sustainable development goals. An expert said, “So far, none of the development programs in our country have given special attention to disaster risk reduction. However, if we want to make progress and improve our disaster management capabilities, we need to allocate a portion of our development programs specifically toward disaster preparedness. By doing so, it becomes logical to allocate a budget for these efforts, and managers would feel obligated to comply with these requirements” (P. 22). Developing educational programs to help promote society’s understanding of risks, focusing on promoting a culture that fosters awareness and understanding of risks at all levels of society, and concentrating on non-governmental organizations (NGOs) to raise societal awareness were other identified solutions in this category.

### Executive actions in the strategy No2: strengthening disaster risk governance to manage disaster risk

This strategy emphasizes that disaster risk governance is vital at international, national, and regional levels in all sectors. The main strategies outlined in this priority are recommendations for governments to implement disaster risk management programs at all levels. The first solution identified was to merge related organizations with similar functions into a single national organization. An expert said, “One of the significant governance challenges we currently face is the presence of multiple organizations with overlapping responsibilities. This leads to confusion and a lack of accountability during disasters. It is proposed to merge similar organizations and establish a unified mechanism” (P-3). The establishment of regional risk governance laws and regulations and the development of implementation and facilitation measures for these laws was another issue addressed in this context. The experts emphasized that the existence of laws alone is not enough for risk management, so necessary executive guarantees should be considered. A participant said, “Laws should be formulated based on risk assessment and the needs of that area. The next point is the lack of an executive guarantee. In some places, for example, there are construction regulations, but there is no supervision of their correct implementation” (P-5). Strengthening risk management’s position in governance documents by including it in 5-year plans was another solution identified in this category. An expert said, “When formulating macro-level plans for a country, it is common practice to refer to high-level documents. However, a specific document like Sendai may not always be used as a reference for macro-level documents. When the macro document is designed based on a document like Sendai, its operational plans are automatically aligned in that direction” (P-30). Another solution was the Stakeholder analysis to get approval, offer dependable funding, and improve the collaborative strategy of stakeholder organizations. An expert said, “Failing to identify stakeholders and address their specific needs and demands can lead to the failure of risk management programs. It is crucial to consider and incorporate their perspectives in the planning phase to increase the chances of success and mitigate potential risks” (P-11). Establishing an information network at the national level for distributing information to relevant entities was another solution identified in this category. A participant said, “The decision-making process relies on information. This concern becomes more complex in disaster risk management as incomplete or inaccurate information can potentially lead to disasters. An integrated system that effectively processes and provides decision-makers with comprehensive information can significantly reduce the likelihood of decision-making errors” (P- 19). Another solution in this field was to compile requirements for obtaining a risk management position following the meritocracy principle. An expert said, “Defining qualification conditions in risk management is a crucial step towards ensuring that only individuals with appropriate education and experience are appointed to relevant positions. By setting requirements, organizations can establish a structured framework that helps prevent appointing individuals who lack the necessary expertise or knowledge” (P-1). Another said, “I believe that management stability is crucial to disaster risk management. A competent person is hired for a post related to disaster risk management, but before their plans can be implemented, the government changes and the manager is replaced. This results in a cycle of starting over, and the problem remains unresolved” (P-3). Another solution was performing comparative research to discover a model for the country and standardize rules and procedures. The experts in this field emphasized the need to use the model as a guide for the country’s overall disaster risk management system. An expert said, “One of our country’s challenges is the absence of a comprehensive and indigenous model tailored to our specific needs. While we can draw inspiration from models of other countries, implementing them verbatim is not feasible. Because unique economic, cultural, and contextual factors in our country are not reflected in the patterns of other nations” (P-14). Another solution of this strategy involved developing a comprehensive risk management plan, implementing continuous national monitoring, and shifting focus towards program-oriented approaches rather than individualistic ones. The experts in this field emphasized that the disaster risk management plan should be written so that it is not specific to one government and has an implementation requirement for any government at any time. One of the participants said, “A crucial point is that risk reduction programs should not be dependent on a specific government. The next government should not say, “I won’t implement this program”; instead, it should strive to improve its weaknesses by updating the program.” (P-24). Increasing the proportion of funds for risk reduction in the national macro budget was another identified solution in this field. Experts believed that efficient and effective programs should have an acceptable budget at the macro level. An expert said, “If you aim to implement earthquake risk reduction measures in an area, the costs associated with demolishing or renovating houses will require financial resources. On a macro scale, these costs will be considerably higher. However, it’s a matter of acknowledging that if we aspire to reduce risks, we must allocate the necessary funds for it” (P-7). Attention to comprehensive risk management, including risk probability, capacity, and vulnerability was the last solution identified in this field. Experts emphasized the importance of adopting a multidimensional approach. It is crucial to consider other factors that contribute to disasters, such as enhancing capacity and reducing vulnerability. An expert said, “In any location, there will always be a certain level of risk. Therefore, it is crucial to identify the factors that make us vulnerable to specific risks and work on reducing them. Simultaneously, capacity building is essential, as societies evolve, and their needs naturally change over time.” (P-9).

### Executive actions in the strategy No3: investing in disaster risk reduction for resilience

The strategy highlights investing in structural and non-structural retrofitting as a key element in improving community resilience. These actions are crucial for saving lives, preventing and minimizing damage, and ensuring an efficient response and recovery. The strategy identifies 8 solutions, which are explained below:

The first solution identified in this strategy is to utilize legislative tools, such as tax breaks and loan grants, to encourage individuals to invest in this sector. According to experts, the involvement of the general public is one of the most influential factors in this field. An expert said, “The best programs will not succeed unless they attract and engage people’s cooperation. To attract people’s opinions, it is important to consider the cultural context of the region. Offering various incentives can also help individuals feel they are part of the program.” (P-32). Another solution in this field is to provide training to managers and community influencers to promote investment in disaster risk reduction (DRR). According to experts, the lack of attention and investment in DRR is partly due to the lack of education. This training should be provided to both managers and the general public. An expert said, “The lack of attention from individuals and managers towards investing in risk reduction can be attributed, in part, to the lack of education at the community level. We must be honest with society and explain the risks. On the other hand, we should also communicate that investing in reducing these risks now can protect your property and life from future disasters.” (P-4). Incorporating DRR projects into current construction and development programs and allocating funds from the National Development Fund, was another solution identified in this strategy. According to experts, developing countries are vulnerable to major disasters due to their limited resources. The occurrence of disasters can delay development plans for several years. “A participant suggested that every year, a percentage of the country’s income be deposited into the National Development Fund. This fund is reserved for the future development of the country’s core industries and critical infrastructure. However, it is better to allocate a certain percentage of it annually as a necessary expense for investing in risk reduction. This way, the level of risk will gradually decrease, and if a disaster occurs, the fund will not be depleted”. (P-12). Experts have proposed conducting comparative research to leverage global experiences regarding the effects of investing in DRR and improving access to technical and transparent disaster information. They have also emphasized the importance of conducting comparative studies after an accident to assess the impact of investments. A participant said, “Why don’t we conduct a comparative study to examine a leading country in this field? We could analyze their risk level, the amount they invested to mitigate risk, the extent to which the risk level was reduced, and ultimately, the cost-effectiveness of their efforts.” (P-2). Developing knowledge-based companies in the field of DRR and investing in them is another solution identified in this strategy. An expert said, “Sometimes, an expert should come forward and state that these hazards exist in society with a certain probability, and that the cure for them is also available. Another expert should then explain in detail the potential harm to society if a certain risk occurs, as well as the associated cost of preventing it. It would be great if all the opinions and solutions of experts could be collected and organized by a knowledge-based company” (P-16). Another solution was to enact legislation for the investment of economic enterprises, polluting industries, and insurance in DRR, as well as to update building regulations. A participant said, “One of the issues that we have paid less attention to in disaster risk management is the crucial issue of insurance. Insurance can significantly alleviate the financial burden on the government in the event of a disaster. On the other hand, if people are convinced that by paying a small amount, they can prevent significant financial loss and even loss of life, they will be more likely to participate.” (P- 31). Another expert has suggested that, “In addition to current laws, a new law should be enacted, which mandates that large and polluting industries allocate a portion of their income and profits towards mitigating risks.” (P-3). Another solution in this strategy was to prevent the advertising of harmful products and services and promote the purchase of safe products, personal safety equipment, and other necessary safety and protection equipment by the general public. One of the participants said “I am very surprised that an expert would come to the media and talk about the harm of a product. Then the same media advertises this product. Which one should the audience believe? We should identify goods or services that pose a risk to people and the environment and prohibit their advertisement. (P- 16). The final executive action in this field was to adopt legislation that compels governments to invest in DRR and facilitates the participation of other sectors, such as NGOs, private and charitable institutions, and international organizations. According to experts, the presence of unnecessary laws, as well as the absence of necessary laws, can impact risk management. A participant said, “In my opinion, the legislature should pass a law that accomplishes two important objectives. Governments will need to invest in risk reduction. The next step is to pass a law that encourages the involvement of others.” (P-28).

### Executive actions in the strategy No4: enhancing disaster preparedness for effective response and to “Build Back Better” in recovery, rehabilitation and reconstruction

Although the Sendai Framework emphasizes risk reduction, it is necessary to allocate a portion of the program to preparedness for response and recovery. In the implementation of this strategy, seven solutions were identified and are explained below.

Establishing an up-to-date early warning system and designing community-based early warning protocols are the first solutions identified in the preparedness improvement strategy. According to experts, it is necessary to enhance early warning systems based on the risks and requirements of the region. A participant said, “In the last few years, we have witnessed the significant impact of not having an accurate and timely early warning system on flood management. This system is inefficient. The outdated technology, based on obsolete analysis models, is unable to provide timely and accurate information. This system makes so many mistakes that people no longer believe in it” (P-29). The enhancement and improvement of the Incident Command System (ICS) to facilitate and synchronize efforts among various agencies tasked with disaster response was identified as an additional measure within this strategy. One of the participants said, “It is not correct to define the same normal organizational structure as an incident command system. Perhaps the head of the organization is not the right person to lead that operation and someone else should take charge of the incident. This issue requires training and practice so that everyone can understand why the structure changes at that particular moment.” (P-8). Compilation of a comprehensive disaster preparedness plan and practicing the program at regular intervals was another executive action. The experts emphasized that it is not always possible to use fixed preparation programs. They stated that risk assessments should be conducted periodically, and preparedness plans should be developed based on the assessment findings. An expert said, “Before we write a preparedness and exercise plan, it is important to conduct a risk assessment and determine the priorities. We should then act accordingly based on the results. After some time, those risks may no longer be a priority and new risks may emerge. If we do not pay attention to this issue, our resources will be completely wasted and we will be unable to meet the needs of the affected population.” (P-11). Another executive action identified in this field was the preparation and implementation of training programs for disaster preparedness and response, tailored to different target groups ranging from elementary schools to households and universities. An expert said, “Many of the behaviors that people exhibit during disasters are natural human reactions for survival, some of which are completely wrong, such as running and fleeing during an earthquake. When you want to change this behavior, you have to train. The education program should also be tailored to suit different age groups.” (P- 17). Paying special attention to the role of social networks and media in transmitting timely and accurate information during disasters, as well as providing proper education to the public, was proposed in this strategy. The experts believe that exaggerating unimportant issues or, conversely, downplaying major actions can significantly alter the course of events. An expert said, “Unfortunately, we neglected the role of virtual social networks.” These networks have both positive and negative aspects. We have to work a lot on improving the quality of our training programs, as they offer extensive coverage and can provide excellent training opportunities to people. It may even provide the basis for rumors or the overthrow of governments, so this foundation should be examined more carefully.” (P-32). Another solution was to improve information systems and technologies, including simulation and GIS, to develop risk maps, forecast disasters, and manage logistics. A participant said, “We need a robust infrastructure in the field of disaster detection and response. This infrastructure must be capable of processing information in real-time, providing a map, identifying high-risk areas, and ensuring that its information is comparable across different time periods.” (P-7). The last action involved utilizing NGOs, the private sector and international organizations to enhance societal preparedness. In this solution, experts emphasized that non-governmental groups, particularly community-based organizations, have a much closer relationship with individuals and a better understanding of their needs. Additionally, these groups use language that is more relatable to the people who speak a similar language. One of the experts said, “We have to accept that sometimes, for various reasons, people do not adhere to government statements. Here, people have a stronger inclination towards community-based and non-governmental organizations because they feel a sense of ownership and belonging to them. So, we can provide inclusive education programs to improve preparedness for the largest possible population.” (P- 2).

We will discuss the results in the following.

## Discussion

### The strategy of recognizing and understanding the risk of disasters

This priority highlights the need to recognize the risk of disaster in all of its aspects, including vulnerability, capacity, exposure, assets, hazard characteristics, and environment. Based on the study findings, paying attention to two points is very important. First, determining and identifying risks based on scientific methods. Second, how to share information on identifying risks among society and interest groups. Information is the basis of decision-making, and people make decisions based on their perception of the received information. A more rational decision will be made if the information is based on reality and provided to the right person correctly. In their study, Bennett et al. state that natural hazards are a common catalyst for promoting disaster risk perception. Therefore, understanding the current and future risk scenarios requires a detailed initial assessment of existing risks and risks at local and national levels. The outputs from the first assessment provide significant results of local hazards so that appropriate policies and practices enable disaster risk managers to focus on areas of high sensitivity and vulnerability [6]. Aven states that quantitative assessments can only provide a snapshot of risk for managers and decision-makers, while decision-makers must look beyond risk description. Therefore, it is necessary to carry out a risk assessment using scientific methods and by experts. In this way, judgments take a scientific basis, reducing the possibility of errors in decisions [3]. In their study, Surianto et al. state that several necessary steps should be taken to improve disaster risk understanding. These measures include the political will to enhance the understanding of risk in society, increasing the budget with the approach of understanding the risk, conducting detailed and scientific assessments of several risks in different regions, planning based on the existing risks, using scientific evidence to improve the understanding of the society’s risk, and finally, developing policies and laws related to risk perception. In their study, they state that disaster risk management measures focused more on preparedness and response in the past, but now, more sustainable approaches have moved toward mitigation and recovery. Therefore, it is necessary to understand the existing risks [46]. DuHadway et al. state that decisions in the four stages of disaster risk management are based on a correct understanding of risks. Accordingly, an integrated system is required that provides timely information to relevant people. In this way, time and resources can be managed appropriately as two critical factors in disaster management. Their study emphasizes that establishing or developing information infrastructures and using new technologies such as IT is beneficial in this field. When risk monitoring and data analysis are done by experts and shared among different layers of society, it can be ensured that decisions will have a scientific basis [10]. Settembre-Blundo et al. believe that if disaster risk management items are integrated into an organization’s development program, it will increase risk perception because all levels of the organization, from management to personnel, are forced to adjust their goals based on them [42]. Kelly et al. state that when people in the community or officials need a correct understanding of the risks due to the lack of awareness of the severity or consequences of the risk, the probability of engaging in risky behaviors is much higher. Therefore, using different tools for different target groups is necessary to help improve their understanding of existing risks. These tools can include individual and specialized training for managers or public training for members of society [19]. Liu et al. have studied the role of culture in risk perception and disaster risk management. In their study, they state that both national and organizational cultures influence people’s behavior toward risks. National and local culture mainly refers to popular beliefs, and organizational culture refers to the organization’s work and information flow. They emphasize that changing the culture will be crucial to changing risk-averse societies [20]. As observed, the researches conducted in the field of risk perception confirm the findings of the present study. Although many studies have examined only one or two aspects of risk perception, this study identified precious solutions using a qualitative method. In this way, it is possible to improve the understanding of the risk of disasters in developing countries by relying on these strategies.

### The strategy to strengthen governance and leadership for disaster risk management

Risk governance at the national, regional, and global levels is essential for effective and efficient disaster risk management. This purpose requires a clear vision, plans, guidelines, inter-sectoral coordination, and stakeholder participation. Strengthening risk governance is essential for prevention, mitigation, preparedness, response, recovery, encouraging inter-agency partnership mechanisms and sustainable development. In their study, Pilli-Sihvola and colleagues emphasize that the existence of parallel structures at the national level leads to inefficiency in governance and policy implementation. According to them, the development of horizontal structures in disaster risk reduction programs and adaptation to the region’s risks before the policy formulation process can prevent the occurrence of inefficiency. They suggest merging similar organizations and taking responsibility for one organization is more effective [37]. Faivre et al. introduce an integrated approach to improve risk governance. They believe that the existence of similar and parallel organizations leads to a waste of resources [11]. In their study, Surianto et al. state that disaster risk reduction programs should be integrated into all sectors, including national, regional, and local, to strengthen risk governance. In addition, regulations and public policies should include the roles and duties of the public and private sectors and include the following: Integrating disaster risk management programs into government services and infrastructure, providing incentives for the actions of individuals, families, communities, and businesses, promoting mechanisms and initiatives for the community, including financial incentives, educational and public awareness initiatives, reporting requirements, and legal and administrative measures and finally, creating coordination and organizational structures [46]. Ruiz-Rivera et al. state that local governments, through some actions, can fulfill their governance role in disaster risk management, leading to its promotion. These measures include policy formulation in disaster risk reduction, professional human resources, adequate budget, multiple risk assessment and integrated planning, risk assessment from different areas, and updating [40]. Tozier emphasizes that in Asian countries, local authorities play a vital role in planning regional development, improving the safety of schools and students, encouraging citizens and villagers to make retrofits against disasters, and promoting and developing community-based support networks. Therefore, they should pay special attention to strengthening disaster risk governance [49]. Manda et al. also state in their study that local governments are responsible for the safety of their citizens and communities, and more than a complementary role in overcoming risk management challenges is needed. Local governments should identify and classify the roles and responsibilities of stakeholders through a careful assessment and then delegate responsibilities. In this way, local governance and national governance in risk management are strengthened [21]. Matsuoka et al. state that the identification and analysis of stakeholders are essential to promote risk governance. Due to their nature, disasters have multiple responders, and naturally, different stakeholders from the national, local, and community levels are involved. When other stakeholders support a particular program, it can increase its acceptability [24]. Faivre et al. state that creating a national information network is vital for the real-time monitoring of risks. For example, due to European climate change, this system can monitor the ecosystem in real-time and provide the necessary information for decision-makers. This information plays a vital role in risk management and government decisions [11]. In their study, Munene et al. introduced the ability of countries to adapt to existing conditions as a fundamental principle for disaster risk governance. They state that countries should extract different models for different conditions by conducting comparative studies and adapting them to their situations. In this way, they can choose the appropriate model to reduce the risk based on the existing disasters [33]. Djalante et al. also emphasize the importance of local governments promoting risk reduction programs. According to them, the lack of cooperation and coordination of local governments in planning, budgeting, and other measures can lead to the failure of programs in implementation. In addition, they suggest that these programs become national and international laws to mandate risk reduction programs. In this case, the laws will be independent of the governments and every government must comply with them in any period [9]. In their study, Mol et al. suggest increasing budgets and credits for risk reduction measures. They also emphasize that compulsory insurance against regional disasters can lead to the strengthening of risk governance [30]. Ullah et al. emphasize in their study that more is needed to assess the risks alone to achieve sustainable cities. Comprehensive risk management is necessary to achieve this goal. In this way, in addition to evaluating the probability of occurrence of risks, vulnerability, and capacities should also be calculated [50]. The findings of other studies confirm the results of the present study. Most of the studies emphasized that the most critical issue in strengthening disaster risk governance for the success of the Sendai Framework and its ultimate goal of reducing disaster risk is the role of communities, especially local communities, as the primary stakeholders. Programs focusing on communities can also better recognize the resources available in regions and be more successful in reducing disaster risk.

### Strategies for investing in disaster risk reduction to build resilience

Generally, investment is associated with concepts such as profit and market terms. To earn profit and increase their income, people invest part of their resources in different fields and benefit from their growth. This issue is more complicated in disaster risk reduction because, in many cases, there is no visible profit like in other economic sectors. In addition to critical infrastructure, large-scale disasters can also affect the property and resources of people and society and cause irreparable damage. The existence of global trade centers worldwide with extensive supply chain networks has made the impact of disasters no longer limited by national borders, and widespread damage in one region can spread to other areas and countries. In their study, Mizutori et al. consider the use of legal incentives as a way to attract investment in disaster risk reduction. They state that people, especially the private sector, should be motivated to invest. Methods such as reducing or waiving taxes, granting loans, granting subsidies, etc., will be attractive solutions for them [27]. Millar et al. state that one of the main challenges in disaster risk management is transforming a scientific concept into a government policy. The scientific language is often complicated for operational managers and makes them face decision-making errors. Therefore, avoiding complex language in persuading managers to invest is necessary. Another critical point in the investment in disaster risk reduction is uncertainty in the future. Environmental conditions in the future may be very different from the present. In this way, none of the planning approaches can provide a general answer to all problems. In such a situation, managers prefer to take short-term measures instead of long-term ones whose effects could be more apparent to show their management time worth. The result of these actions will be investment deviation and waste of resources. The solution to this issue is to encourage flexibility in planning approaches. In this way, if the situation suddenly changes, the program can be quickly modified [26]. Hoffman et al. state in their study that investments in disaster risk reduction must be made to achieve sustainable cities. They emphasize that one of the appropriate methods for this purpose is integrating disaster risk reduction into construction programs. This will lead to building codes, and people and governments must invest in them [15]. Ahmed states in his study that between 2006 and 2012, Indonesia’s investment in disaster risk reduction increased from 2.6 to 10 trillion rupiahs. More than two-thirds of this investment has been allocated to. The sectors of mitigation, improving preparedness, research, and education. Ahmad believes that training in the field of disaster risk reduction is necessary for both people and officials. When people understand potential disaster losses, they may be motivated to invest in risk reduction. The critical point in this study is that despite allocating a significant budget to risk reduction, there needs to be a law on how to spend it, which is a weakness of this program. With defining the cost lines, resources may be spent on necessary things. In this study, compared to Indonesia, the country of Pakistan has been examined, and the results show that the approach of disaster risk management programs in this country is mainly response-oriented, and mitigation measures are insignificant [1]. Weichselgartner et al. emphasize the importance of paying attention to the lessons learned from previous disasters in several aspects. First, by studying the past, it is possible to identify the types of regional disasters and their frequency and Repetition Period. So people can be sensitized to the hazards in their place of residence. Second, by reviewing the statistics of casualties and damages from past incidents, people can be encouraged to invest in retrofitting. Finally, by checking the experiences of other countries and the way to manage the risk of disasters, it is possible to define suitable models for the target communities [52]. In his study, Shaw points out that part of the lack of safety in societies is related to unsafe goods produced by industries. According to Shaw, more than convincing people to invest in retrofitting is needed. If people use dangerous products for retrofitting, the expected results will certainly not be achieved, and the level of risk in society will stay the same. Therefore, by enacting laws, governments must prevent the manufacture, advertisement, and distribution of unsafe goods. The study also suggested that polluting industries could be forced to invest in disaster risk reduction. In this way, they will continuously seek to reduce their environmental risks [44]. Surianto et al. emphasized that in addition to the duty of governments in investing to reduce the risk of disasters, facilitating laws should also lead to increased cooperation and attracting private sector investment. They state that the private sector makes more than 70% of investments in the world, and in this way, ensuring the security of these investments is very important because, in case of disasters, both natural and man-made, regional, national, and local economies will be affected. It is predicted that the role of the private sector in the world economy will increase in the future, and for this reason, an active role has been assigned to the private sector in reducing the risk of disasters [46]. As it was observed, the findings of the conducted studies strengthen the results of the present study. Convincing people and managers to invest in disaster risk reduction is the most critical issue in this field. On the one hand, there are managers, decision-makers, and budget officials who may show resistance to investment. On the other hand, in developing societies where the per capita income is much lower than in developed countries, it is much more challenging to carry out risk-reduction activities. In these societies, high inflation leads to not investing in options with intangible benefits.

### Strategies to promote disaster preparedness for effective response and recovery

The steady growth of disaster risk, including the increase in the number of people and assets at risk, with the lessons learned from the occurrence of disasters, indicates the need to strengthen disaster preparedness, use models to predict disasters, integrate disaster risk reduction into preparedness and assurance of improved response capacity and effective recovery. In their study, Moradi et al. state that retrofitting critical infrastructures such as hospitals in developing countries are essential due to the high degree of vulnerability and limited resources. They emphasize that in such communities if retrofitting is not done before disasters occur, huge costs are needed to respond to disasters. In addition, establishing and updating early warning systems can play a vital role in improving preparedness [31]. Banwell et al. emphasize that upgrading early warning systems is one of the ways to increase preparedness against disasters, especially weather disasters. They state that climate change and the increasing importance of weather hazards and early warning systems can significantly improve response and recovery [4]. Mohanty et al. also emphasize that early warning systems should be understandable to communities and compatible with their culture. For this purpose, it is essential to develop educational programs for the community to learn how to warn and take action after the warning [29]. In their study, Farcas et al. have stated the development and strengthening of the Incident Command System (ICS) as a solution to improve preparedness. Considering the COVID-19 pandemic, they emphasize that the existence of this system can lead to a more appropriate response when disasters occur, especially unknown disasters [13]. In their study, Shah et al. emphasize that to reduce the risk of disasters in more traditional and response-based societies, developing comprehensive preparedness programs can partially eliminate the decision-making gaps in the pre-event stages. These programs should be designed based on the needs of society, identify major risks at the level of society, and create a detailed plan for preparedness and response to them. In addition, this program should pay special attention to local conditions, including social, ethnic, economic, political, and cultural characteristics [43]. In their study, Mariam et al. state that developing educational programs based on target groups is necessary to improve knowledge, attitude, and preparedness against disasters. According to them, to change their behavior in disasters, their knowledge and attitude must first be changed with proper training, and then the correct behavior should be replaced. In addition, the same programs will not work for all society. Different groups live in a society that has different needs, and their level of understanding is completely different [23]. Mano et al. state that risk communication comprises a significant portion of the scientific literature on preparedness. Most risk communication focuses on various tools and measures at-risk populations use to receive, access, and use the information for warning and preparedness. They emphasize that using social media at the individual and organizational levels can contribute to disaster risk reduction programs. The content produced in this field should be different and based on the needs of the target population. Using the same messages for the population can reduce its effectiveness. Therefore, to improve preparedness through social media, the target groups should first be identified, and then the content should be designed and published via social media [22]. In their study, Sakurai et al. discussed the importance of information systems for disaster preparedness. They state that there needs to be a comprehensive view of information technology use in all disaster management phases. Information systems play a fundamental role in recording, exchanging, and processing information. They emphasize that these systems play a vital role in preventing disasters or their effects and promoting preparedness. If real-time information is quickly processed and provided to the user, it can reduce the adverse effects of the disaster [41]. In his study, Baytiyeh states that it is necessary to pay attention to the role of groups outside the government to improve preparedness against disasters because they can have a role in influencing society. The author highlights the vital role of religiopolitical organizations in influencing socio-cultural factors and contributing to implementing DRR. He emphasizes that not paying attention to these organizations, especially in developing countries, can lead to the failure of disaster risk reduction activities [5]. Various studies confirm research findings and show how preparedness can be improved. There are many studies in the field of preparedness, and in them, practical and specific actions are mainly emphasized, which we will not mention due to limitations. In general, disasters have shown that planning for response and recovery should be done before disasters occur because by integrating disaster risk reduction in development measures, promoting retrofitting and increasing community resilience, much better opportunities for response and recovery can be provided. At first glance, preparedness and recovery may be different from the original goal of the Sendai document to disaster risk reduction, but it should be noted that from the moment each accident occurs, actions can be taken to prevent the next one, and for this reason, it is necessary pay special attention to preparedness, response and recovery.

## Conclusion

The Sendai Framework, which is designed to reduce disaster risk, focuses on the following four strategies: Understanding disaster risk, strengthening disaster risk governance to manage disaster risk, investing in disaster risk reduction for resilience, and enhancing disaster preparedness for effective response and “Build Back Better” in recovery, rehabilitation, and reconstruction. However, this framework has yet to provide any definite implementation solutions. It has only made suggestions because the economic, social, political, and cultural conditions and the level of development of the countries are different. This study attempted to investigate the solutions for implementing the four strategies in Iran as a developing country by using an in-depth qualitative study with experts and policymakers in disaster risk management. The study’s findings can be used as a guide for other developing countries. In this study, various solutions were presented for each of the priorities of the Sendai framework. Each country can design comprehensive plans based on the development level, needs, and resources. On the other hand, by reviewing the identified solutions, one can understand the challenges in developing countries because the solutions are expressed in response to a lack and challenge. Therefore, by referring to the challenges and solutions, the conditions of such communities can also be considered in future universal plans.

### Limitations

The biggest limitation of qualitative studies is the difficulty of access to experts to participate in the study. In this study, an effort was made to gather all relevant experts from all over Iran at the Kerman University of Medical Sciences and discuss the issue. The next limitation occurred during the interviews. Considering that some of the experts had important management positions, it was difficult to access them. Therefore, some interviews took months. Another limitation was the coincidence of the study with the Covid-19 pandemic, which made it somewhat difficult to hold meetings. The participants were assured that the sessions would be conducted in hygienic conditions.

## Data Availability

The datasets generated and/or analyzed during the current study are available from corresponding author.
